# Autoimmune haemolytic anaemia associated with epstein barr virus infection as a severe late complication after kidney transplantation and successful treatment with rituximab: case report

**DOI:** 10.1186/s12882-015-0096-3

**Published:** 2015-07-18

**Authors:** Alexander J Hamilton, Lynsey H Webb, Jennifer K Williams, Richard J D’Souza, Loretta SP Ngu, Jason Moore

**Affiliations:** Department of Nephrology, Royal Devon and Exeter NHS Foundation Trust, Exeter, UK; Institute of Biomedical and Clinical Science, University of Exeter Medical School, Exeter, UK; Department of Haematology, Royal Devon and Exeter NHS Foundation Trust, Exeter, UK

**Keywords:** EBV, Kidney transplantation, AIHA, Rituximab

## Abstract

**Background:**

Autoimmune haemolytic anaemia (AIHA) is a rare complication following kidney transplantation and usually occurs early in its course. It is characterised by autoantibodies or alloantibodies directed against red blood cells (RBCs).

**Case presentation:**

We describe a 44 year old woman who presented 5 years after kidney transplantation with profound transfusion dependent warm AIHA. Investigations confirmed an IgG autoantibody against RBCs and high titre Epstein-Barr virus (EBV) viraemia. The patient was at higher risk for EBV disease being seronegative at the time of transplantation but had detectable EBV capsid IgG antibody at the time of presentation. The haemolysis was refractory to high dose steroid and intravenous immunoglobulin. There was a rapid and complete resolution of both the anaemia and the viraemia following rituximab therapy, with no adverse events. Twenty-six units of blood were required during the course of treatment.

**Conclusions:**

To our knowledge this is the first reported case of EBV associated AIHA in a renal transplant recipient. It highlights a rare pathology associated with post-transplant EBV infection, of broad interest to transplant physicians, haematologists, and microbiologists, and the effective novel use of monoclonal anti-CD20 therapy.

**Electronic supplementary material:**

The online version of this article (doi:10.1186/s12882-015-0096-3) contains supplementary material, which is available to authorized users.

## Background

Autoimmune haemolytic anaemia (AIHA) is an immune disease characterised by antibodies directed against autologous red blood cells (RBCs). Typically patients exhibit anaemia with reticulocytosis, spherocytes and polychromasia on the blood film with a positive direct antiglobulin test (DAT) which is the hallmark [1], in addition there is often increased unconjugated serum bilirubin and elevated serum lactate dehydrogenase (LDH). The antibodies can be subdivided into “warm” or “cold” agglutinins depending on the thermal range of activity [2] and the subsequent anaemia can be profound and life threatening leading to large transfusion requirements. The aetiology is unknown, categorised either as primary (idiopathic) or secondary when associated with malignancy (in particular chronic lymphocytic leukaemia), connective tissue and inflammatory diseases, infections (both viral and mycoplasma associated with a cold AIHA) [2, 3], or drugs (e.g. purine analogues and alkylating agents) [4, 5]. Alloantibody can also lead to haemolysis post haematopoietic stem cell transplant, solid organ transplant (i.e. passenger B lymphocyte syndrome), pregnancy and after transfusion [6].

Epstein-Barr virus is one of the eight human herpes viruses, and common in humans. In the United States, by the age of 40 as many as 95 % of adults have been infected with EBV. Infants (after maternal antibody protection has disappeared) and children have asymptomatic or mild disease. In adolescence or young adults, EBV causes infectious mononucleosis in up to 50 %. Symptoms of infectious mononucleosis are commonly fever, sore throat, and lymphadenopathy and are almost never fatal. EBV then establishes a lifelong dormant infection in B cells [7]. It is a carcinogenic virus associated with Burkitt’s lymphoma, Hodgkin’s disease and nasopharyngeal carcinoma [8]. After transplantation EBV disease can present with varied manifestations, including nonspecific febrile illness, gastroenteritis, hepatitis, mimicking other viral infections, and most seriously post-transplant lymphoproliferative disorder (PTLD) [9]. It remains unclear why EBV causes autoimmune disease. IgM antibodies to autoantigens are normally present in the plasma at low non-pathologic titre. It has been suggested that the B-cell clones that normally produce these autoantibodies are altered to produce IgG antibodies in high and pathogenic levels in AIHA [10]. Alternatively, defective control of IgG auto reactivity by autologous IgM [11] or altered T-cell function [12] has been proposed.

AIHA after solid organ transplantation has been reported infrequently [13] and usually occurs early in the course [14]. In this report, we describe a severe and late presentation in the context of donor associated EBV viraemia, the treatment and use of novel therapy.

## Case presentation

In January 2013 a 44 year old white female presented to our institution with malaise, exertional breathlessness, night sweats and a headache. She had received a pre-emptive deceased donor kidney transplant 5 years earlier. The kidney characteristics were donation after circulatory death [DCD], human leucocyte antigen (HLA) mismatch A1:B1:DR1, cytomegalovirus (CMV) serology of both donor and recipient were negative but Epstein-Barr virus (EBV) serology of the donor was positive whereas the recipient was negative. The aetiology of end stage renal disease was likely congenital, initial presentation was with advanced chronic kidney disease and a single functioning kidney. The only baseline comorbidity was congenital nystagmus. Primary graft function was excellent and stabilised with serum creatinine 90 μmol/L [1.0 mg/dL] and eGFR 60 ml/min/1.73 m^2^. Induction immunosuppression was with basiliximab and maintenance was tacrolimus, mycophenolate mofetil and prednisolone, as per local protocols. She had been treated with valganciclovir for CMV viraemia at 3 months and mycophenolate was electively withdrawn in line with institutional protocol after 7 months. The patient continued on dual immunosuppressive therapy thereafter and there were no episodes of acute rejection. Otherwise she had an uneventful first 5 years except for cervical intraepithelial neoplasia grade 1 and acne rosacea.

**Fig. 1:**
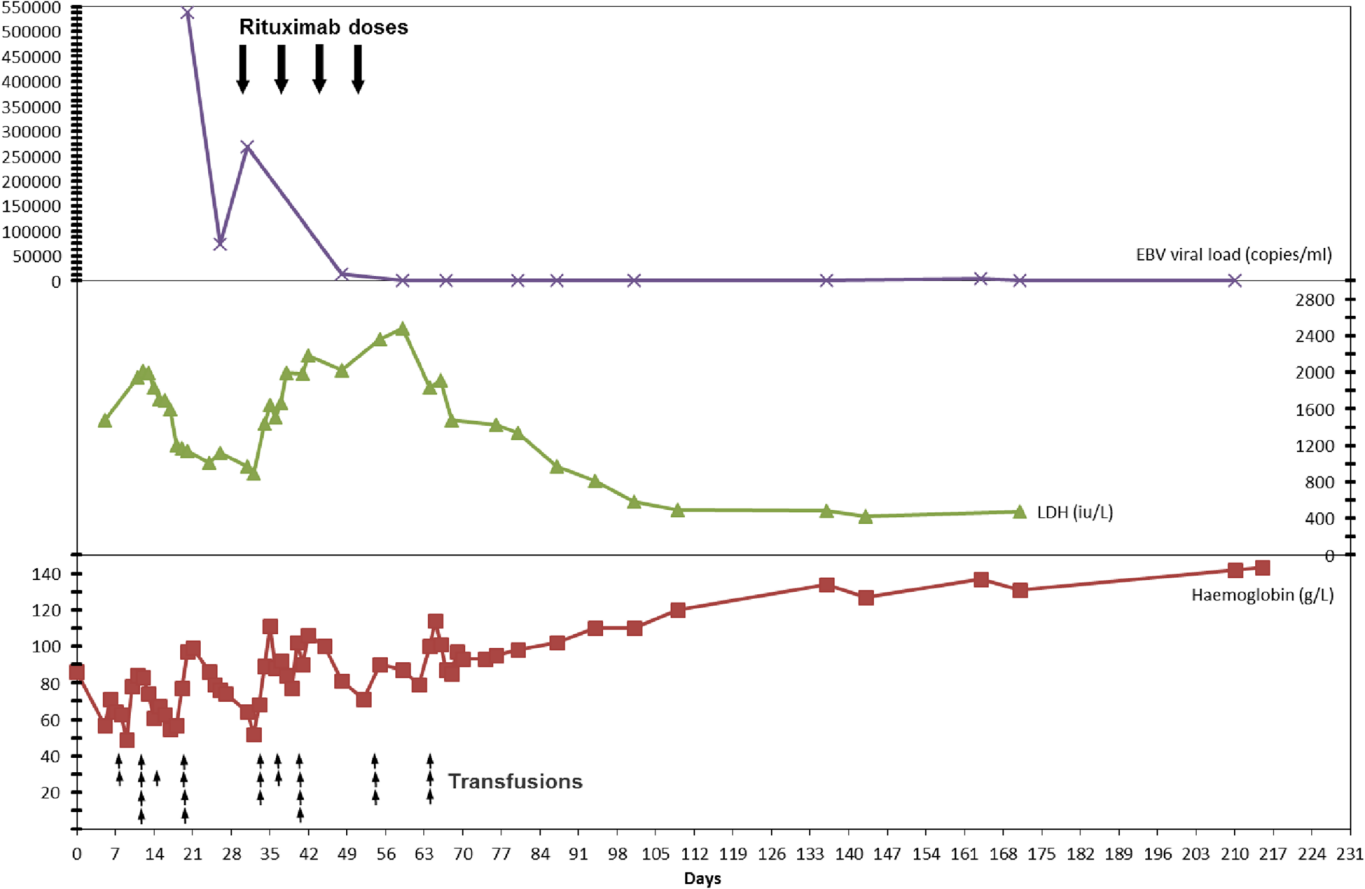
Serial haemoglobin, LDH and EBV viral load and response to Rituximab therapy

On examination she was pale, jaundiced and tachycardic, with dark urine. There was no lymphadenopathy. All investigations are shown in Additional file 1: Table S1. They demonstrated a severe anaemia (haemoglobin 57 g/L) with parameters consistent with haemolysis. DAT was positive confirming a warm agglutinin AIHA. She required blood transfusion support and was initially treated with high dose prednisolone (1 mg/kg) with little effect. A 5 day course of intravenous immunoglobulin (0.4 g/kg/day) was started on day 9. At this point a high serum titre of EBV (538355 copies/ml) was detected with serology suggesting this was reactivation from previous exposure to donor virus and not a primary infection (see Additional file 1: Table S1). She went on to have a total body CT scan which detected no abnormal lymphadenopathy and a bone marrow aspirate and trephine was consistent with haemolysis and no evidence of a lymphoproliferative disorder. Tacrolimus was converted to sirolimus on day 28. After an initial fall in serum EBV load there was a deterioration in clinical state with increasing transfusion requirements followed by an increase in EBV load. The patient was given rituximab 375 mg/m^2^ on day 30, followed by a further three weekly doses. At the beginning of the treatment course she had received 16 units of blood and the EBV viral load was 268649 copies/ml. After the fourth dose of rituximab there was a significant decline in transfusion requirement and the EBV viral load had fallen to undetectable levels. Seven transfusions were required during the rituximab course and three following the course. Her serial haemoglobin, LDH, EBV viral load, rituximab doses and transfusions are shown in Fig. 1. She had achieved complete remission 2 weeks after starting the rituximab. There was no deleterious effect on graft function and no adverse drug events were noted. In total she required 26 units of blood with significant iron overload, the serum ferritin rose to 2850 μg/L which had fallen to 950 μg/L 12 months after remission. Despite the acute illness graft function was unchanged and she remains well on prednisolone and sirolimus immunosuppression with no recurrence of EBV viraemia or anaemia.

## Discussion

In this case severe haemolysis secondary to a warm agglutinin IgG antibody presented five years after kidney transplantation and was associated with a high titre EBV viraemia. Although the association between EBV presenting as infectious mononucleosis and AIHA is well established in the general population [7] surprisingly this has not been reported after kidney transplantation. Our patient had no evidence of lymphadenopathy. The serological investigations of a positive anti-EBV capsid IgG antibody in our patient would suggest exposure more than three months prior to presentation and the patient was at higher risk with a negative serostatus at the time of transplantation. It is very possible that the patient had been asymptomatically viraemic earlier in the transplant course but we would not have detected this with no protocol for nucleic acid testing (NAT) more than one year post transplantation. A recent report suggests mycophenolate use post transplantation may well be associated with less EBV DNAaemia and our patient was not taking an anti-proliferative agent [15].

There were clinical concerns in our case about the lack of response to initial therapy, with steroids and intravenous immunoglobulin, and the possibility of a developing PTLD, therefore conversion to sirolimus and rituximab treatment was undertaken. Sirolimus rescue for tacrolimus-associated post-transplant autoimmune haemolytic anaemia has been successfully reported [16], and the evidence base is growing for its use in PTLD and post-transplant malignancy [17, 18]. Multiple case studies and retrospective reports have indicated success with use of the monoclonal anti-CD20 antibody rituximab in both adults and children with resistant idiopathic AIHA and EBV associated AIHA post haemopoietic stem cell transplantation [19]. In the latter the mechanism of action is felt to be the elimination of EBV infected B cells. The effectiveness of this strategy in our case was dramatic and well tolerated.

The adverse effects of multiple transfusions in this case are yet to be quantified but they are potentially very significant due to iron overload and HLA sensitisation. Clearly the earlier the haemolysis is stopped the better. International guidelines focus on EBV monitoring (NAT) in the first year post transplantation, KDIGO recommend monthly monitoring for high risk individuals (seronegative) from 3–6 months (the most common period for primary EBV infection), then every 3 months up to 1 year with reduction of immunosuppression when titres rise [20]. Based upon a single case report, we do not believe the long term monitoring of all seronegative/high risk individuals to be an effective or practical strategy in the avoidance of such a scenario. The absence of this presentation from the transplant literature to date indicates it is likely to be a rare phenomenon but the effective safe intervention of rituximab warrants awareness.

For the practising nephrologist, this case is important in highlighting the potential for a belated, life-threatening situation associated with a common virus exposed to the patient following the act of deceased donor kidney transplantation; most importantly it demonstrates the failure of conventional first line therapy leading to the rapid successful use of a novel treatment.

## Conclusion

To our knowledge this is the first report of AIHA associated with EBV viraemia post kidney transplantation. The AIHA was unresponsive to conventional management, however full resolution of haemolysis and viraemia was achieved with rituximab treatment most likely by eliminating the EBV infected B cells.

## Consent

Written informed consent was obtained from the patient for publication of this Case report and any accompanying images. A copy of the written consent is available for review by the Editor of this journal.

## Additional file

Additional file 1: Table S1. Summary of baseline investigations with laboratory reference ranges shown in brackets. Abbreviations: MCV, mean cell volume; LDH, lactate dehydrogenase; Ig, immunoglobulin; CFT, complement fixation test; CMV, cytomegalovirus; PCR, polymerase chain reaction.

## Abbreviations

AIHA: Autoimmune haemolytic anaemia
RBC: Red blood cell
EBV: Epstein-Barr virus
DAT: Direct antiglobulin
LDH: Lactate dehydrogenase
DCD: Donation after circulatory death
HLA: Human leucocyte antigen
CMV: Cytomegalovirus
eGFR: Estimated glomerular filtration rate
CT: Computer tomography
Ig: Immunoglobulin
NAT: Nucleic acid
PTLD: Post transplant lymphoproliferative disorder
