# Supplementary material for: Autoimmune haemolytic anaemia associated with epstein barr virus infection as a severe late complication after kidney transplantation and successful treatment with rituximab: case report
Source: BMC Nephrol. 2015 Jul 18;16:108. doi: 10.1186/s12882-015-0096-3 (PMC4506635; doi:10.1186/s12882-015-0096-3)
Supplement: Additional file 1: Table S1. — Summary of baseline investigations with laboratory reference ranges shown in brackets. Abbreviations: MCV, mean cell volume; LDH, lactate dehydrogenase; Ig, immunoglobulin; CFT, complement fixation test; CMV, cytomegalovirus; PCR, polymerase chain reaction. [file 12882_2015_96_MOESM1_ESM.docx]

Additional file 1: Table S1. Summary of baseline investigations with laboratory reference ranges shown in brackets.

Abbreviations: MCV, mean cell volume; LDH, lactate dehydrogenase; Ig, immunoglobulin; CFT, complement fixation test; CMV, cytomegalovirus; PCR, polymerase chain reaction.

| **Investigation** | **Result and units** | **Reference range** |
| --- | --- | --- |
| Haemoglobin | 57 g/L | 120-160 |
| White blood cells | 8.9 10^9^/L | 3.6-11.0 |
| Platelets | 179 10^9^/L | 150-400 |
| Unconjugated bilirubin | 30 umol/L | <16 |
| MCV | 111.2 fL | 82-98 |
| Reticulocytes | 195 10^9^/L | 50-100 |
| LDH | 1472 iu/L | 240-490 |
| B12 | 862 ng/L | >180 |
| Folate | 11.3 ug/L | 4.6-18.7 |
| Creatinine | 103 umol/L | 44-80 |
| Urea | 10.1 mmol/L | 2.5-7.8 |
| Tacrolimus | 5.6 ug/L | 5.0-20.0 |
| Alanine amino transferase | 31 iu/L | 10-36 |
| Alkaline phosphatase | 57 iu/L | 30-130 |
| Albumin | 43 g/L | 35-50 |
| Complement C3 | 1.27 g/L | 0.75-1.65 |
| Complement C4 | 0.24 g/L | 0.14-0.54 |
| IgA | 1.32 g/L | 0.8-2.8 |
| IgG | 6.1 g/L | 6.0-16.0 |
| IgM | 0.34 g/L | 0.5-1.9 |
| Mycoplasma pneumoniae Ab (CFT) | <1/10 | |
| CMV PCR | Not detected | |
| BK virus PCR | Not detected | |
| Parvovirus B19 PCR | Not detected | |
| Blood film | Infrequent spherocytes with polychromasia | |
| Direct Coombs Test | Direct antiglobulin test - moderately positive (IgG coating) | |
| Bone marrow aspirate/trephine  Immunophenotyping | Erythroid hyperplasia with reversed myeloid:erythroid ratio  Immunochemistry stain negative for CD20 | |
| CT head/thorax/abdomen/pelvis | No radiological evidence of significant lymphadenopathy | |
| EBV Nuclear antigen antibody  EBV Capsid IgG antibody  EBV Capsid IgM antibody | Negative  Positive  Negative | |
